# Silicone Foley's catheter: A useful splint in ear surgeries

**DOI:** 10.4103/0970-0358.41111

**Published:** 2008

**Authors:** Siddharth K. Karanth, Nitin J. Mokal

**Affiliations:** Department of Plastic and Reconstructive Surgery and Burns, Bai Jerbai Wadia Hospital for Children, Parel, Mumbai - 400 012, Maharashtra, India

**Keywords:** External auditory meatal stenosis, microtia, silicone Foley's catheter

## Abstract

Maintenance of ear projection and post auricular sulcus in staged ear reconstruction in microtia is a trying problem. So also is the maintenance of the patency of the external auditory meatus following recanalization and meatoplasty. Numerous splints and dressing techniques have been described for the above situations. Some of the problems encountered include the availability of the materials, cost, expertise in fabrication and compliance.

**Aims:**

To devise a simple, reliable, inexpensive and readily available splint for the maintenance of post auricular sulcus and external auditory meatus opening.

**Settings and Design:**

A silicone catheter is made out of a soft and inert material that doesn't cause tissue necrosis or any loss of skin graft. The basic design is that of a simple, self-retaining type of splint that doesn't dislodge and can be prepared within minutes on the operating table.

**Materials and Methods:**

This splint has been used in four cases of microtia reconstruction and one case of congenital external auditory meatus stenosis between June 2006 and August 2007. A 14 or 16 Fr silicone Foley's catheter was used. The proximal end of a catheter of required length was retained and the distal part was cut off. The catheter was looped into a circle around the base of the reconstructed ear and secured in position with a suture. A similar construct was used in cases of external auditory meatus reconstruction or recanalization. The funnel-shaped distal drainage end was sutured to the circular frame near the region of the tragus. This funnel was inserted into the external auditory canal.

**Results:**

The catheter was found to sit snugly in the newly created sulcus, thereby maintaining the sulcus and ear projection. It aided in maintaining the meatal opening of a satisfactory diameter in the case of external auditory canal recanalization. It was never found to slip or get dislodged in any of the cases. There was no skin graft loss or tissue necrosis due to the use of the splint.

**Conclusions:**

The silicone Foley's catheter is found to be a simple, readily available, inexpensive and reliable self-retaining splint following ear elevation in microtia and external auditory meatus recanalization. The catheter is easily constructed and applied intraoperatively. The results following its usage have been uniformly good in all cases without causing any adverse events at the operated site or discomfort to the patient ensuring good compliance.

## INTRODUCTION

The maintenance of the ear projection and postauricular sulcus in cases of ear reconstruction in microtia is a known problem. This situation may require splints to be applied and worn for a long duration of time to counteract the contracting forces of the skin-grafted, post auricular tissue.[1–5] Thermoplastic splints are often used for post auricular sulcus or as ear guards.[1,4] These splints are difficult to retain and some need to be harnessed around the head.[1–3] In cases of stenosis of the external auditory meatus and the adjoining external auditory canal, recurrent stenosis is an impending problem following recanalization and meatoplasty. Splintage is required to maintain patency. Numerous splints and dressing techniques have been described.[6–10] Problems encounterd include the availability of the materials, cost, compliance and expertise in fabrication. Some of these splints are cumbersome, making compliance a problem.

## MATERIALS AND METHODS

This method of splintage has been used for four cases in the third stage of ear reconstruction in microtia, and in one case requiring correction of a stenosed external auditory meatus.

**Figure 1:**
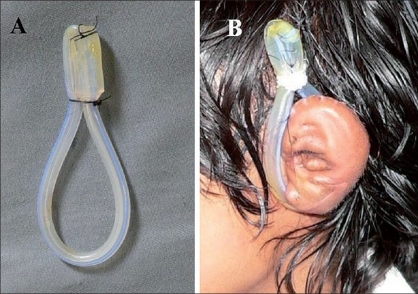
(A) Shows the splint made from the silicon Foley's catheter which was cut and sutured to itself. (B) Shows the splint in position in a case of microtia after ear elevation

The silicone Foley's catheter is a readily available commodity. It is made of an inert, nontoxic, soft and pliant material. The catheter is inexpensive and easily afforded by most patients. The catheters are also of different sizes making variations in splintage easy. The splint was fabricated using the catheter of 14 or 16 Fr size. Construction was quick, easily made on-table and applied immediately. The proximal end of the catheter of required length was retained and the distal part bearing the port was cut off. The catheter was looped into a circle around the base of the reconstructed ear and sutured to itself in the region of the upper pole of the ear [Figure 1].

In each of the cases, once the ear was elevated, the retro auricular area was covered with the flap from the mastoid fascia over a piece costochondral cartilage and a split thickness skin graft was applied. This splint was applied over tulle gras covering the skin graft and the rest of the dressing was given over this. The first dressing was done on the fifth postoperative day - the catheter-splint was removed, cleaned and reapplied over a tulle gras. Once the graft take was complete, the dressings were abandoned and only the splint was applied for three to six months.

**Figure 2:**
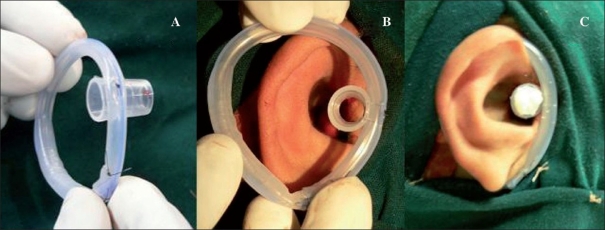
(A) Shows the splint made from silicone Foley's catheter with the funnel-shaped drainage end sutured to the splint. (B) Shows the orientation of the splint while applying with the funnel-shaped attachment being placed near the tragus. (C) Shows the splint in position with a small pack in the lumen of the funnel

A similar construct was used in the one case of congenital meatal stenosis of the external auditory canal. In addition to the circular loop of the catheter around the ear, the funnel-shaped distal end (not the port to fill the balloon) was sutured to this circular frame near the region of the tragus [Figure 2]. This modification of the splint was used after meatoplasty was performed with a local flap and split thickness skin grafting. The funnel was inserted into the recanalised meatus and external auditory canal lined with tulle gras. The splint was applied intraoperatively and continued for six months. The two described splints were applied continuously even when the patient was asleep or physically active and were removed only while bathing.

## RESULTS

**Figure 3:**
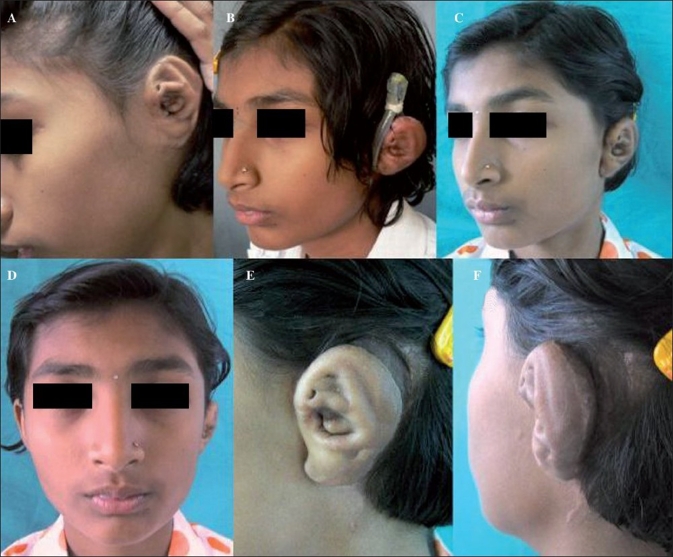
(A) Shows the representative case of left microtia prior to ear framework elevation. (B) Shows the splint in postion two months following surgery. (C-F) Are four month follow-up images of the same patient with good projection and a well-formed retroauricular sulcus

**Figure 4:**
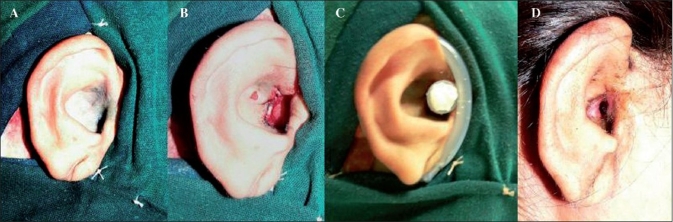
(A) The preoperative image of the case of congenital meatal stenosis of right ear. (B) Is the intraoperative image of the reconstructed meatus and adjoining auditory canal. (C) Shows the splint in position. (D) Shows the postoperative image four months after the surgery with a wide meatus and patent auditory canal

The patients with microtia reconstruction had good postoperative ear projection with a well-maintained retroauricular sulcus. There was no skin graft loss, necrosis or infection in any of the cases. The splints were well-tolerated and compliance was good. The patients wore them for a period ranging from three to six months [Figure 3]. In the congenital meatal stenosis case, the patient was comfortable and compliant with the splintage. There was no graft loss or tissue necrosis seen in this case. The follow-up was for a period of eight months and the reconstructed meatus was patent and of sufficient diameter with no restenosis [Figure 4].

## DISCUSSION

The reconstruction of the ear in a case of microtia is one of the most challenging problems in plastic surgery. The final outcome is assessed by the symmetry in the size and projection of the ear, the adequacy of the contour of the ear and an adequate retro auricular sulcus. The problems related to the loss of projection of the ear and the obliteration of retro auricular sulcus is well-known. [1–4] Numerous splints and ear guards have been described to avoid the above-mentioned complications. [1–5] The splint described here serves not only to maintain the ear projection but also to preserve the retro auricular sulcus which has a tendency to contract over a period of time.[4] The splint has the advantage of being self-retaining without the need for any straps or harnesses around the head or chin. The splint is inexpensive, easily available and can be made within minutes on-table and applied immediately. This move avoids the need for tie-over dressings and makes the patient accustomed to the splint early in the postoperative phase. The splint is soft, inert and nontoxic and has not been found to cause any local tissue reactions or graft loss or necrosis. The splint is easily concealed by the hair. All these factors help improve compliance to the splintage.

Meatal stenosis is another trying problem faced by otorhinolarygologists and plastic surgeons. Restenosis is a major problem in these cases with restenosis rates varying from 10-33%.[5,11] Numerous splints using rubber tubes, silicone tubes, thermoplastic materials and dressing techniques have been described in literature to avert this problem.[6–10] We have found that a splint made from silicone Foley's catheter is useful in this setting too.

The funnel-shaped attachment is soft and cone-shaped conforming to the shape of the cartilaginous part of the external auditory canal. The funnel is neither too soft nor too rigid, thus, it does not cause any harm to the flaps or the skin grafts at the meatus or canal and at the same time, maintains the luminal diameter to the required size. As the funnel is hollow, it allows drainage of external auditory canal and does not interfere with hearing. It also shares the same advantages of affordability, availability, compliance and the lack of need for any special expertise or equipments to make it.

## CONCLUSION

The silicone catheter-splint is a useful splint in cases of ear reconstruction in microtia and following meatoplasty. In the former, it has been found to maintain ear projection and retroauricular sulcus with acceptable long-term results. In the case of meatal stenosis, the patency following surgery was maintained with the use of the splint. The splint is easily available, inexpensive, easy to construct and has good patient compliance.
